# Gene Expression Profile in Similar Tissues Using Transcriptome Sequencing Data of Whole-Body Horse Skeletal Muscle

**DOI:** 10.3390/genes11111359

**Published:** 2020-11-17

**Authors:** Ho-Yeon Lee, Jae-Yoon Kim, Kyoung Hyoun Kim, Seongmun Jeong, Youngbum Cho, Namshin Kim

**Affiliations:** 1 Genome Editing Research Center, Korea Research Institute of Bioscience and Biotechnology, Daejeon 34141, Korea; hylee@kribb.re.kr (H.-Y.L.); jyoon@kribb.re.kr (J.-Y.K.); kekgoo@gmail.com (K.H.K.); lovemun@kribb.re.kr (S.J.); yycho@kribb.re.kr (Y.C.); 2 Department of Bioinformatics, KRIBB School of Bioscience, University of Science and Technology (UST), Daejeon 34141, Korea

**Keywords:** RNA-Seq, skeletal muscle, differentially expressed genes

## Abstract

Horses have been studied for exercise function rather than food production, unlike most livestock. Therefore, the role and characteristics of tissue landscapes are critically understudied, except for certain muscles used in exercise-related studies. In the present study, we compared RNA-Seq data from 18 Jeju horse skeletal muscles to identify differentially expressed genes (DEGs) between tissues that have similar functions and to characterize these differences. We identified DEGs between different muscles using pairwise differential expression (DE) analyses of tissue transcriptome expression data and classified the samples using the expression values of those genes. Each tissue was largely classified into two groups and their subgroups by k-means clustering, and the DEGs identified in comparison between each group were analyzed by functional/pathway level using gene set enrichment analysis and gene level, confirming the expression of significant genes. As a result of the analysis, the differences in metabolic properties like glycolysis, oxidative phosphorylation, and exercise adaptation of the groups were detected. The results demonstrated that the biochemical and anatomical features of a wide range of muscle tissues in horses could be determined through transcriptome expression analysis, and provided proof-of-concept data demonstrating that RNA-Seq analysis can be used to classify and study in-depth differences between tissues with similar properties.

## 1. Introduction

Genetic studies of livestock have primarily been aimed at increasing production. Most livestock animals raised today are for meat, and improvements have been made to control fat content and muscle properties to induce rapid muscle growth or maximize the favored types of tissues [1,2,3,4,5,6].

Horses have been identified as a suitable model for studying gene expression in skeletal muscle related to exercise ability [7,8,9,10,11], which is rare for livestock. The present study is similar to a prior report of muscle activity and associated genes in humans [12,13,14,15]. Skeletal muscles have different physiological characteristics depending on their role, which is related to the composition of muscle fibers according to the main purpose of the muscle in question.

The primary component that determines the physiological function of muscles is myosin heavy chain (MyHC), which is myofibril’s thick filament. MyHC is classified into three types, type I, type IIa, and type IIb/IIx, depending on their contraction rate and metabolic phenotype. Type I is a slow oxidative (SO) fiber that has a slow contraction speed and obtains ATP from oxidative phosphorylation (OXPHOS). Type IIb/IIx is a fast glycolytic muscle (FG) that contracts rapidly and take ATP from glycolysis, and type IIa is a fast-oxidative-glycolytic (FOG) fiber with intermediate properties of type I and type IIb/IIx.Type I is a slow oxidative (SO) fiber that has a slow contraction speed and obtains energy from OXPHOS.

Higher content of type I fibers is associated with slower contraction and highly oxidative metabolism. These slow-twitch muscles are reddish due to the high mitochondria, myoglobin, and capillary distribution. The force that can be created by muscle contraction is not strong, but their fatigue resistance makes them used for posture maintenance or continuous and repetitive activities. On the contrary, a higher content of type II (b) fibers is associated with faster contraction and a a glycolytic phenotype [16,17]. Fast-twitch muscles such as these produce ATP through anaerobic glycolysis, and they can exert a strong force in a short time due to their fast-contraction speed but become fatigued easily. Because they use less oxygen, they contain fewer mitochondria, blood vessels, and myoglobin. Consequently, they are less reddish than slow-twitch muscle and look beige or pale-colored. MyHC fibers vary in composition depending on the muscle’s use, and their ratio can be changed by muscle conditioning. Additionally, depending on the characteristics of muscles altered by MyHC content, gene expression associated with the corresponding functions will differ.

In general, to evaluate the distribution, or ratio, of each fiber, measurement of protein expression, or comparison of cross-section thickness to identify the fiber types via microscopy is used [7,16]. However, next-generation sequencing (NGS) analysis of RNA-Seq data can reveal the dominant characteristics of each tissue by comparing global gene expression between tissues, even though the actual fiber composition cannot be directly identified. In addition, it is also possible to compare one tissue repeatedly with tissues from different sites [18].

Most studies of equine muscle tissue are focused on the analysis and improvement of exercise function. The primary breeds used in these studies have been Thoroughbred and Arabian horses, which are used primarily for horse racing or riding, and only a few tissues, such as the blood or gluteus medius, have been evaluated [7,8,9,10,11]. In the present study, we analyzed transcriptome sequencing data from 18 different skeletal muscle tissues taken from the Jeju horse. The Jeju horse is a type of pony that lives on Jeju Island in Korea. It is ~110–120 cm in height and resilient to disease [19].

However, we could not study the unique characteristics of Jeju horse itself because a sufficient number of samples were not secured. Furthermore, because of the specificity of finely classified muscle data from the whole body, we could not obtain similar data from previous studies. Thus, we aimed to identify the transcription expression between skeletal muscle tissues by utilizing the particularity of the subdivided tissue data.

**Figure 1:**
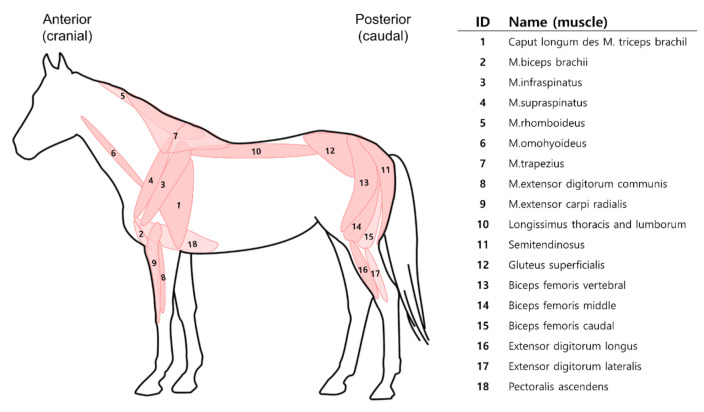
Name and location of 18 skeletal muscle tissues of *Equus caballus*.

RNA-Seq data of 18 different muscle regions of the Jeju horse were subjected to differential expression (DE) analysis (Figure 1) [20,21], which was classified by k-mean clustering. We used RNA-Seq data to determine if the detailed classification of skeletal muscles with similar properties ware possible. Additionally, we sought to determine the physiological characteristics of each muscle compared with similar and proximal tissues by identifying specific gene expression patterns between tissues.

## 2. Materials and Methods

### 2.1. Ethics Statements

All animal experiments were performed in accordance with the guidelines of the Institutional Animal Care and Use Committee and were approved by the Animal Genomics and Bioinformatics Division, National Institute of Animal Science (No.2014-080). All efforts were made to minimize animal suffering.

### 2.2. Sample Collection

The specimens were obtained from a male horse, born on 12 June 2012, managed by the National Institute of Animal Science, R.D.A, Jeju, South Korea. It was slaughtered through exsanguination after electric stunning. Each tissue was collected from 18 regions of skeletal muscles in a hot-carcass state and immediately frozen to liquid nitrogen and preserved at −80 °C.

### 2.3. RNA Sequencing

Samples were obtained from a Jeju horse, a region-specific horse breed in Jeju, South Korea. Sample libraries from 18 different skeletal muscles were obtained from one Jeju horse. Ribosomal RNA was removed from total RNA using a RiboMinus Eukaryote kit for RNA-Seq (Thermo Fisher Scientific, Sunnyvale, CA, USA). The RNA-Seq library was prepared using a TruSeq RNA kit (Illumina; San Diego, CA, USA). Sampling and RNA sequencing were conducted by the National Institute of Animal Science, Rural Development Administration, and sequenced using an Illumina HiSeq 2000 (2 × 101 bp). The GEO accession number for this data set is GSE113147.

### 2.4. Data Processing

Quality assessment was conducted using FastQC version 0.11.5 software (https://www.bioinformatics.babraham.ac.uk/projects/fastqc) to analyze sequence reads in the fastq file format. Using the NGSQCToolkit [22] with a Phred quality score < 20 and less than 50 bp in total length were removed with paired-read and adapter sequences were trimmed.

The reference RNA sequence FASTA files were mapped to the horse genome (EquCab2 79, Equus_caballus.EquCab2.DNA.chromosome.1~31, X, MT, nonchromosomal.fa) downloaded from the Ensembl FTP database. The alignment was performed using the STAR (v 2.5.2b) RNA-Seq aligner with a two-pass method [23]. Using this approach, the first alignment detected splice junctions based on transcript information, and the final alignment was performed using splice junctions as a guide. As a guide for transcript alignment, we used the relevant gtf file (Equus_caballus.EquCab2.87.gtf) as a reference. The alignment process provided read counts for a total of 26,841 genes in 18 samples.

### 2.5. Differential Expression Analysis

First, the differentially expressed genes (DEGs) between each Jeju horse skeletal muscle were identified by pairwise analysis using the negative binomial test of the DESeq R package [24]. Based on the reads per gene counts identified through sequence mapping, the pairwise DEGs (*p*-value < 0.05) of all 18 tissues were identified. All DEGs (*n* = 1292) identified by pairwise analysis and their read counts were used to implement clustering of samples by their differential gene expression.

Clustering was performed using the hclust and the k-means function of the R package, and the optimal k value (k = 2) was calculated by the NbClust package in the R. The k-means clustering divides 18 tissues into large two groups, and we subdivided two groups into three subgroups each. Based on the classified cluster information, we conducted a DE analysis between groups using entire genes (*n* = 26,841). DESeq2 was used for DE analysis [25].

### 2.6. IPA Analysis

Further analysis to determine the functions of the identified DEGs was conducted using Ingenuity Pathway Analysis (IPA, Ingenuity Systems, Redwood, CA, USA) [26]. IPA was used to conduct enrichment analysis of the canonical pathway of the gene set and the inter-molecule network. To import the gene list and avoid omissions in the advanced analysis results as much as possible, the Ensembl Equus Caballus gene ID was converted into a well-annotated Ensembl human ID to identify DEGs.

### 2.7. Gene Ontology

Gene ontology (GO) enrichment analysis of DEGs was performed using the biological processes of the GeneOntology site (http://geneontology.org/, Powered by PANTHER) [27]. GO terms selected only the term of the main category, except subcategories.

## 3. Results

### 3.1. Read Alignments and Results

By mapping the raw data to the ensemble horse genome (EquCab2 79) using STAR-2PASS alignment, the raw reads were aligned to a total of 26,841 genes. The number of raw sequence reads per sample ranged from 55.7 to 83.1 M, with an average of 64.1 M and an average input read length of 202 bp (2 × 101 bp). Uniquely mapped reads averaged 53.1 M, representing 83.2% of the total average reads.

### 3.2. Differential Expression Analysis

#### 3.2.1. Classification

**Figure 2:**
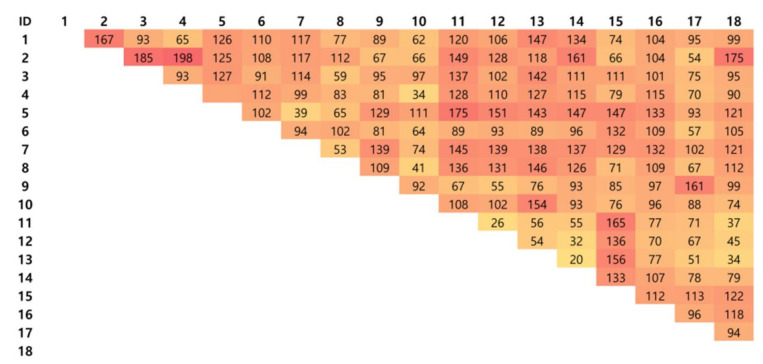
Number of DEGs (Differentially expressed genes). Number of DEGs per tissues identified through pairwise tests of 18 skeletal muscles (min = 20, max = 198), the sample ID is the same as shown in Figure 1.

A total of 1292 DEGs were identified through pairwise analysis. This number means that most of the DEGs shown in Figure 2 are overlapping. These are key features that can explain the difference between skeletal muscles among 26,841 genes prepared for clustering 18 samples. We classified samples into hierarchical clustering (using the value of log2 (count + 1)) and k-means clustering through the raw count data of these 1292 DEGs. The optimal k value calculated using the NbClust was set as 2 (Supplementary Materials Figure S1).

**Figure 3:**
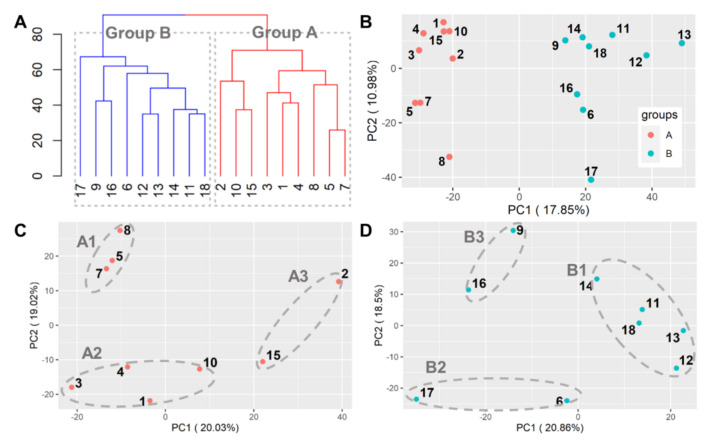
Classification of samples by gene counts. The results of k-means clustering are shown on hierarchical clustering and PCA (Principal component analysis) plot. (**A**) The result of hierarchical clustering using read counts of total DEGs was the same as K-means clustering (k = 2). (**B**) Clustering results for all 18 groups on PCA plot. (**C**) Subgroups in group A by k-means clustering on the PCA plot. (**D**) Subgroups in group B by k-means clustering on the PCA plot.

Samples were divided primarily into two groups, and the classification results were the same in both hierarchical clustering and k-means clustering (Figure 3). Each group classified by clustering was labeled as “A” or “B” (Figure 3A) and normalized using DESeq2 [25], and subsequently plotted on a PCA plot (Figure 3B). Groups A and B were further divided into subgroups based on the results of k-means clustering (k = 3). The k = 3 is a maximum value that allows two or more tissues to be included in one subgroup for DESeq2 analysis. The clustering results through k-means are presented on the PCA plot of Figure 3B–D.

#### 3.2.2. Comparison of A vs. B

As a result of performing DE analysis on the total gene counts of groups classified into A and B using DESeq2, a total of 1264 DEGs (up = 597, down = 667, adjusted-*p* < 0.05, |log2Foldchange| > 0.58 (FC = 1.5)) were identified. The expression fold-change for A vs. B in DESeq2 results represents the value of A/B. Subsequent “upregulated/downregulated” means higher/lower expression in the former’s condition of A vs. B. Genes in the Ensemble gene id format were converted into HGNC gene symbol format for further analysis, and a total of 857 (up = 487, down = 370) were successfully converted.

The IPA and gene ontology were used for gene set enrichment analysis of the identified DEGs. Table 1 shows the pathways within the top 10 scores (–log (*p*-value); > 1.3) with *z*-score values (not 0) in the IPA canonical pathway results.

GO analysis was performed to elucidate the functional enrichment of upregulated DEGs of biological processes using PANTHER. Table S1 contains the top 10 GO terms based on the fold enrichment value. Directly related to the function of skeletal muscles are “regulation of muscle adaptation (GO:0043502)” and “regulation of striated muscle contraction (GO:0006942)”.

#### 3.2.3. Group A

To identify the characteristics of the muscles in each group, we conducted DE for subgroups in groups A and B, respectively. In A and B, a total of three sets of 1 vs. 2, 1 vs. 3, and 2 vs. 3 were performed. In comparison of group A1 and A2, a total of 236 DEGs (up = 128, down = 108, adjust-P <0.05, |log2FC| > 1) were identified, of which 200 genes (up = 101, down = 99) were converted to the human gene symbol. As a result of analyzing the DEGs by IPA (Table 2), an increase in the glycolysis and gluconeogenesis canonical pathway was confirmed. This is similar in GO (Table S2), as we could find fast-twitch glycolytic muscle-related GO terms like “positive regulation of fast-twitch skeletal muscle fiber contraction (GO:0031448)”, “canonical glycolysis (GO:0061621)”, and “gluconeogenesis (GO:0006094)” in GO analysis of upregulated DEGs. Considering these results, the muscles of group A1 are more glycolytic than the muscles of A2 and are thought to be closer to the fast twitch.

A total of 383 DEGs (up = 3, down = 380, adjust-*p* < 0.05, |log2FC| > 1) were identified through DE analysis of groups A1 and A3 (301 genes (up = 1, down = 300) were converted to a gene symbol). In A1 vs. A3, all genes were down regulated except three genes, including the only annotated TLE1. The results of the IPA analysis could not identify canonical pathways directly related to metabolic processes, such as glycolysis, and most pathways were associated with immune responses, including T cell signaling pathways (Table 2). Additionally, in GO analysis, which was conducted on downregulated DEGs (Table S3), most of the GO terms related to T cell and immune response were confirmed, and there was no direct result on metabolism.

In group A2 vs. A3, a total of 363 DEGs (up = 98, down = 265, adjust-*p* < 0.05, |log2FC| > 1) were identified. Among them, 283 were converted to human gene symbols, 89 upregulated DEGs, and 194 downregulated DEGs. In the IPA canonical pathway, downregulation of glycolysis was confirmed, and also included a number of immune-related pathways, such as those found in A1 vs. A3 (Table 2). Likewise, in the GO of downregulated DEGs (Table S3), “canonical glycolysis (GO:0061621)” and “gluconeogenesis (GO:0006094)” were identified, and the GO term related to the immune response occupied the majority as in the IPA analysis. GO terms identified in upregulated DEGs (Table S2) include “cardiac myofibril assembly (GO:0055003)”, “detection of calcium ion (GO:0005513)”, “muscle system process (GO:0003012)”, “locomotion (GO:0040011)”, and “movement of a cell or subcellular component (GO:0006928)”.

#### 3.2.4. Group B

The same as group A, organizations classified as group B were also divided into three subgroups by k-means clustering, and DE analysis was conducted for each group. In comparison with group B1 and group B2, a total of 416 DEGs (up = 184, down = 232, adjust-*p* <0.05, |log2Foldchanage| > 1) were identified, of which 355 (up = 148, down = 208) has been converted to human gene symbol. The upregulation of glycolysis, oxidative phosphorylation, and gluconeogenesis was found in the IPA canonical pathway for these genes (Table 3). This suggests that in tissues belonging to group B1, overall energy metabolism occurs more actively than muscles in group B2. This is the same in GO analyzed using upregulated gene (Table S4), “canonical glycolysis (GO:0061621)”, “gluconeogenesis (GO:0006094)”, “regulation of oxidative phosphorylation (GO:0002082)”, “mitochondrial electron transport, NADH to ubiquinone (GO:0006120)”, “aerobic respiration (GO:0009060)”, and other GO terms were also identified.

In group B1 vs. group B3, a total of 219 DEGs (up = 151, down = 68, |log2Foldchanage| > 1) were identified, of which 164 (up = 117, down = 46) were converted to gene symbols. In the IPA analysis, a pathway showing a certain tendency to metabolism was not found (Table 3), and pathways presumed to be upregulated are AMPK signaling, senescence pathway, synaptogenesis signaling pathway, factors promoting cardiogenesis in vertebrates, colorectal cancer metastasis signaling, adrenomedullin signaling pathway, cardiac hypertrophy signaling (enhanced), systemic lupus erythematosus in B cell signaling pathway, neuroinflammation signaling pathway, and hepatic fibrosis signaling pathway were identified. In GO of upregulated DEGs (Table S4), “response to nutrient levels (GO:0031667)”, “regulation of lipid metabolic process (GO:0019216)”, “positive regulation of developmental process (GO:0051094)”, and “positive regulation of multicellular organismal process (GO:0051240)” enrichment was confirmed for, and the GO term indicating a certain metabolism type was not identified.

In the DE of group B2 vs. group B3, a total of 164 DEGs (up = 81, down = 83, |log2Foldchanage| > 1) were identified. Of these, 141 (up = 72, down = 69) were converted to human gene symbols. These DEGs showed downregulation of glycolysis and gluconeogenesis in the IPA canonical pathway (Table 3).

The upregulated DEGs showed only association with “muscle contraction (GO:0006936)” in GO of upregulated DEGs (Table S4), but in analysis using downregulated DEGs (Table S3), they also identified the characteristics of the fast-twitch muscle, such as “positive regulation of fast-twitch skeletal muscle fiber contraction (GO:0031448)”, “canonical glycolysis (GO:0061621)”, and “gluconeogenesis (GO:0006094)”.

## 4. Discussion

In general, it is known that the difference in muscle fiber type and their metabolic properties is the trait that classifies the skeletal muscles. Many studies have demonstrated differences in biochemical-metabolic phenotypes depending on muscle use and fiber composition. In addition, the type and nature of the skeletal muscle fiber that makes up the muscle are transformable and can vary depending on the role the muscle plays. As a result of previous studies of equine exercise capacity, the classification, properties, and related genes of skeletal muscle tissue are well characterized. These prior studies demonstrated a large number of exercise-induced changes, with increased expression of genes associated with oxidative phosphorylation and mitochondrial function in skeletal muscle of individuals endurance-exercised continuously for long periods of time [8,11,15,27]. These results have been verified by molecular biology studies [8,9,15].

In the present study, biochemical analyses using RNA-Seq data from various regions of horse skeletal muscle tissue were conducted, with the objective of identifying whether a classification based on tissue characteristics was possible within very similar tissues. In our 18 skeletal muscle tissue data obtained from Jeju horse, there were no biological or technical replicates for each of the muscle tissues. To compensate for this limitation, the DE between groups was identified by considering the groups of k-means clustering as a replicate for a similar trait.

**Figure 4:**
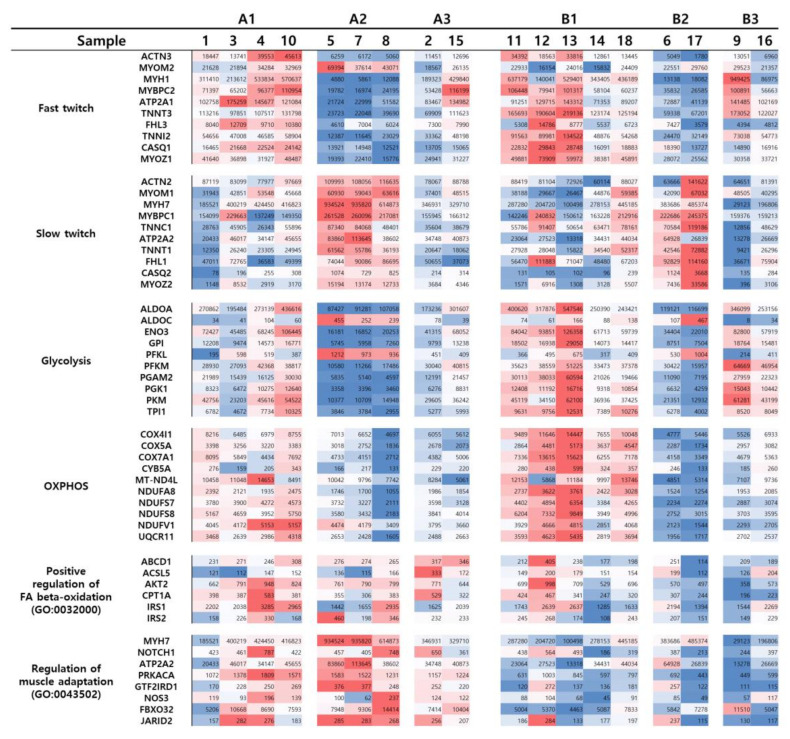
Expression heatmaps in each group and sample for slow-twitch- and fast-twitch-specific genes. The list of genes is based on the (i) fiber-type-specific genes from the literature base (TNNC2 and TNNI1 are not included because they do not have annotation ids in the reference), (ii) DEGs in IPA canonical pathways and GO, (iii) GO database (positive regulation of FA β-oxidation). The color of the heatmap for gene counts is blue for the lower percentile than 50 and red for higher than 50 (for each row).

The DEGs obtained in the pairwise comparison classified 18 muscle tissues into two groups (hierarchical and k-means both), and these were subdivided into three subgroups each. When the expression of genes that determine the fiber type, and the accompanying genes [28], and the expression of genes involved in the functional enrichment of the related traits [29,30,31,32,33] were compared by listing on the basis of clustered samples as a form of the heatmap (Figure 4), it was found that the genes are known to be expressed together with the type of muscle fiber [28], usually increased and decreased here as well. However, the expression of the gene that represents the muscle fiber type and the type of gene that is expected to accompany were not completely consistent.

Among DEGs, the expression of the gene known to be muscle fiber specific also represented various values within the same group A and group B. The difference between the two main groups was more distinguishable in the gene expression of the functional enrichment group of GO. Among the identified DEGs on the heatmap (Figure 4), genes included in “regulation of muscle adaptation (GO:0043502)” were upregulated in group A and downregulated in group B, and genes in the “positive regulation of FA β-oxidation (GO:0032000)” represent similar patterns as well. The functional enrichment for “regulation of muscle adaptation (GO:0043502)” was also identified in the GO results of upregulated DEGs of group A (Table S1). These results suggest that the classification results were preferentially distinguished by differences in muscle adaptation-related functions and gene expressions involved therein.

The canonical pathways identified from group A vs. group B do not directly refer to the effect on skeletal muscle, but many studies have verified their metabolic functions in skeletal muscle and muscle adaptation. As shown in Figure S2, estrogen receptor (ER) signaling exists at the center of functions that DEGs are involved (Table 1). Estrogen is a type of sex hormone that circulates in the body and directly or indirectly affects many molecular pathways through ER, and exercise-induced skeletal muscle adaptation is also affected by ER. ER signaling increases muscle mass regulation and regeneration and is known to enhance ATP production and lipid metabolism by promoting fission fusion and β-oxidation of mitochondria [34,35,36,37,38]. In addition, it was confirmed that a number of canonical pathways included in the table were also affected by the control mechanisms of ER signaling or involved in changes in the types associated with exercise-indicated adaptation [39,40,41,42,43,44,45,46,47,48,49].

In the distribution of muscles shown in Figure 1, the muscles in group A are usually located in the anterior and the muscles in group B are mostly located in the posterior. Taking together these differences in group A vs. group B, this result suggests that the muscles located in the front and rear perform different main functions and thus exhibit different gene expression. Anatomical research of the muscle architecture revealed that the muscle of the forelimbs and hindlimbs have different main roles [21,50]. The proximal horse muscles of the hindlimbs provide energy for locomotion and the muscles of forelimbs act as stiff spring-like struts to support a greater proportion of the body mass [21]. Therefore, the gene expressions for adapting to continuous activity are stronger in the muscles of the forelimbs and anterior that supports the weight of the body. On the other hand, the gene expression for energy generation appears to be more dominant in the muscles of the hindlimbs and the posterior that provide power.

In skeletal muscle adaptation research conducted on porcine, it has been found that adaptation to endurance exercise occurs primarily in forelimb musculature [51]. As shown in Figure 1 and Figure 4, gene expressions for exercise adaptation are dominant in muscles of the anterior and it is thought that the same mechanism will work in equine also. According to Harrison et al. [52], muscle #9 (extensor carpi radialis) located in the forelimbs is activated in the swing state, unlike most of the other muscles in the forelimbs, which are activated in the state of stance. This report could explain why muscle #9 shows a similar expression to the muscles of group B, even though it is located in the anterior.

Unlike general knowledge, the expression of genes directly associated with the skeletal muscle fiber type and with a metabolic tendency (glycolysis, OXPHOS) caused by the fiber type is not unilaterally proportional to the associations of “slow-OXPHOS” and” fast-glycolytic”. It has complex patterns that cannot be explained by a single element. The sub-clusters of groups A and B are sets of tissues that have similarities in these complex expression patterns. The analysis of DEGs between sub-clusters could identify the direct differences of detailed traits, such as fiber-specific gene expression, glycolysis, and oxidative phosphorylation levels (Table 1, Table 2 and Table 3) (Figure 4). Here, the tissues included in the cluster are also mostly located in close or homologous positions (group B3) and are expected to play similar roles. As more tissues are added or replication is secured, more clusters are separated. Thus, differences in simple traits overlap and multiple expression types appear, making it possible to classify a narrower range of locations and functions with only in silico data.

## 5. Conclusions

In this study, we analyzed the function of muscles with gene expression values obtained from transcriptome sequencing data based on in silico data and literature. We identified that RNA-Seq expression data can be used to classify tissues according to their specific characteristics, even among highly similar tissues, such as different skeletal muscles. Skeletal muscles were categorized by their role and difference in gene expression caused by hormonal and cell signaling pathways. The muscles we analyzed were largely classified into two groups by muscle adaptation-related pathways, which reflected their main roles and location. The two groups of tissues classified can once again form clusters with those with similar properties. This sub-cluster is clustered by detailed types of gene expressions, such as fiber types of muscles or energy metabolic pathways.

If there is an opportunity to confirm the tissue composition and protein expression through analyses using classical methods, this would provide an opportunity to verify the results of the present study and to examine the utility of studying animal tissues with NGS analysis.

## Figures and Tables

**Table 1:** Top 10 canonical pathway in IPA analysis of group A vs. group B.

Ingenuity Canonical Pathways	-log (*p*-Value)	*z*-Score	Molecules
Estrogen Receptor Signaling	4.1	1.569	*ADCY1, ADCY5, ADCY9, CACNA1C, CARM1, CREB5, CREBBP, DDX5, FBXO32, FOXO4, GNAZ, GPS2, IGF2R, MAP2K2, MED4, MMP14, MMP15, MMP16, MMP2, NCOR2, NOS3, NOTCH1, PIK3CB, PLCB3, PRKACA, TRIM63*
Endocannabinoid Cancer Inhibition Pathway	3.89	−1.807	*ADCY1, ADCY5, ADCY9, CREB5, CREBBP, DDIT3, MAP2K2, MAP2K7, MMP2, NOS1, NOS2, NOS3, NUPR1, PIK3CB, PRKACA*
Semaphorin Neuronal Repulsive Signaling Pathway	3.48	−0.535	*CSPG4, ITGA3, MAP2K2, MAP2K7, MAPT, PAK4, PDE4A, PIK3CB, PLXNA1, PLXNA2, PLXND1, PRKACA, SEMA3E, SMC3*
GNRH Signaling	3.47	1.941	*ADCY1, ADCY5, ADCY9, CACNA1C, CACNA1G, CACNA1H, CREB5, CREBBP, HBEGF, MAP2K2, MAP2K7, MAP3K11, MMP2, PAK4, PLCB3, PRKACA*
Corticotropin Releasing Hormone Signaling	3.3	1.387	*ADCY1, ADCY5, ADCY9, CACNA1C, CACNA1G, CACNA1H, CREB5, CREBBP, MAP2K2, NOS1, NOS2, NOS3, PRKACA, SLC39A7*
Gαs Signaling	2.93	2.111	*ADCY1, ADCY5, ADCY9, ADD3, ADRB2, CREB5, CREBBP, MAP2K2, PRKACA, RAPGEF2, RYR1*
Spliceosomal Cycle	2.92	−2.646	*DDX46, DHX15, MAGOH, PRPF18, RBM8A, SLU7, ZNF830*
Adrenomedullin signaling pathway	2.86	2.673	*ADCY1, ADCY5, ADCY9, KCNH2, KCNN3, MAP2K2, MAP2K7, MMP2, MYLK2, NOS3, PIK3CB, PLCB3, PRKACA, RAMP2, RXRA, SLC39A7*
White Adipose Tissue Browning Pathway	2.77	2.887	*ADCY1, ADCY5, ADCY9, CACNA1C, CACNA1G, CACNA1H, CREB5, CREBBP, LDHD, PPARA, PRKACA, RXRA*
Calcium Signaling	2.66	1.897	*ATP2A2, CACNA1C, CACNA1G, CACNA1H, CHRNG, CREB5, CREBBP, MICU1, MYH14, MYH7, MYH8, PPP3CB, PRKACA, RCAN1, RYR1, TP63*

**Table 2:** Top 10 IPA canonical pathways in group A.

Ingenuity Canonical Pathways	-log (*p*-Value)	*z*-Score	Molecules
A1 vs. A2			
Glycolysis I	14	1.897	*ALDOA, ALDOC, ENO3, GPI, PFKL, PFKM, PGAM2, PGK1, PKM, TPI1*
Gluconeogenesis I	7.11	1.633	*ALDOA, ALDOC, ENO3, GPI, PGAM2, PGK1*
Calcium Signaling	5.71	1	*ATP2A1, ATP2B2, CASQ2, CREB3L4, MYH1, MYH11, MYL1, MYL6B, TNNI2, TNNT3, TPM1*
Actin Cytoskeleton Signaling	5.47	1.667	*ACTN3, DIAPH3, EGF, FGF9, HRAS, LIMK1, MYH1, MYH11, MYL1, MYL6B, MYLPF*
Protein Kinase A Signaling	4.96	1.265	*CREB3L4, MYL1, MYL6B, MYLPF, NAPEPLD, PGP, PHKB, PLCL1, PLCL2, PPP1R3D, PTPN3, PYGM, TNNI2, UBASH3B*
Estrogen Receptor Signaling	3.84	1.508	*BCL2, CREB3L4, EGF, HRAS, LIMK1, MYL1, MYL6B, MYLPF, PLCL1, PLCL2, SETD7*
Apelin Cardiomyocyte Signaling Pathway	3.65	1.633	*ATP2A1, MYL1, MYL6B, MYLPF, PLCL1, PLCL2*
Synaptic Long Term Potentiation	3.03	0.816	*CREB3L4, HRAS, PLCL1, PLCL2, PPP1R1A, PPP1R3D*
Semaphorin Neuronal Repulsive Signaling Pathway	3.02	0.816	*DPYSL2, LIMK1, MYL1, MYL6B, MYLPF, VCAN*
PAK Signaling	2.8	1.342	*HRAS, LIMK1, MYL1, MYL6B, MYLPF*
A1 vs. A3			
iCOS-iCOSL Signaling in T Helper Cells	24.8	−4.359	*CAMK4, CD247,CD28, CD3D, CD3E, CD3G, CD4, CD80, CD86, FCER1G, HLA-DOA, HLA-DOB, HLA-DRA, ICOS, IKBKE, IL2RA, IL2RB, IL2RG, ITK, LAT, LCK, LCP2, PIK3CG, PTPRC, VAV1, ZAP70*
CD28 Signaling in T Helper Cells	23.8	−3.771	*ARPC1B, CAMK4, CARD11, CD247, CD28, CD3D, CD3E, CD3G, CD4, CD80, CD86, FCER1G, HLA-DOA, HLA-DOB, HLA-DRA, IKBKE, ITK, LAT, LCK, LCP2, PIK3CG, PTPN6, PTPRC, SYK, VAV1, ZAP70*
Th2 Pathway	19.8	−3.638	*CCR1, CD247, CD28, CD3D, CD3E, CD3G, CD4, CD80, CD86, CXCR4, HLA-DOA, HLA-DOB, HLA DRA, ICOS, IKZF1, IL2RA, IL2RB, IL2RG, ITGB2, JAK3, PIK3CG, SPI1, TIMD4, VAV1*
Th1 Pathway	17.2	−3.638	*CD247, CD28, CD3D, CD3E, CD3G, CD4, CD80, CD86, CD8A, CXCR3, HLA-DOA, HLA-DOB, HLA-DRA, ICOS, IL10RA, IL18R1, IRF1, ITGB2, JAK3, PIK3CG, VAV1*
PKCθ Signaling in T Lymphocytes	16.1	−4.69	*CARD11, CD247, CD28, CD3D, CD3E, CD3G, CD4, CD80, CD86, FCER1G, HLA-DOA, HLA-DOB, HLA-DRA, IKBKE, LAT, LCK, LCP2, PIK3CG, RAC2, VAV1, VAV3, ZAP70*
Role of NFAT in Regulation of the Immune Response	15.7	−4.796	*CAMK4, CD247, CD28, CD3D, CD3E, CD3G, CD4, CD80, CD86, FCER1G, FCGR2C, HLA-DOA, HLA-DOB, HLA-DRA, IKBKE, ITK, LAT, LCK, LCP2, PIK3CG, PLCB2, SYK, ZAP70*
Calcium-induced T Lymphocyte Apoptosis	12.9	−3.606	*ATP2A3, CAMK4, CD247, CD3D, CD3E, CD3G, CD4, FCER1G, HLA-DOA, HLA-DOB, HLA-DRA, LCK, PRKCB, ZAP70*
PD-1, PD-L1 cancer immunotherapy pathway	11.1	3.873	*CD247, CD28, CD80, HLA-DOA, HLA-DOB, HLA-DRA, IL2RA, IL2RB, IL2RG, JAK3, LAT, LCK, LCP2, PIK3CG, ZAP70*
Type I Diabetes Mellitus Signaling	10.8	−2.828	*CASP8, CD247, CD28, CD3D, CD3E, CD3G, CD80, CD86, FCER1G, HLA-DOA, HLA-DOB, HLA-DRA, IKBKE, IRF1, PRF1*
B Cell Receptor Signaling	10.4	−3.051	*APBB1IP, CAMK4, DAPP1, FCGR2C, IGHE, IGHG4, IGHM, IKBKE, PIK3AP1, PIK3CG, PRKCB, PTK2B, PTPN6, PTPRC, RAC2, SYK, VAV1, VAV3*
A2 vs. A3			
iCOS-iCOSL Signaling in T Helper Cells	12.3	−3.464	*CD3D, CD3E, CD4, FCER1G, HLA-DOA, ICOS, IL2RA, IL2RG, ITK, LCK, LCP2, PIK3CD, PIK3CG, PTPRC, VAV1, ZAP70*
B Cell Receptor Signaling	10.8	−2.496	*CD22, CREB3L4, DAPP1, FCGR2C, IGHE, IGHG4, IGHM, MAP2K6, PIK3AP1, PIK3CD, PIK3CG, PLCG2, PRKCB, PTPRC, RAC2, RASSF5, SYNJ2, VAV1*
Phospholipase C Signaling	10.1	−2.138	*CD3D, CD3E, CREB3L4, FCER1G, FCGR2C, IGHG4, ITGA4, ITK, LCK, LCP2, MYL2, MYL6B, MYLPF, NAPEPLD, PLCB2, PLCG2, PLD4, PRKCB, RAC2, ZAP70*
Glycolysis I	9.21	−1.414	*ALDOA, ALDOC, ENO3, PFKL, PFKM, PGAM2, PGK1, PKM*
Actin Cytoskeleton Signaling	8.71	−1.604	*EGF, FGD3, FGF9, ITGA4, LIMK1, MYH1, MYH10, MYL2, MYL6B, MYLPF, NCKAP1L, PIK3CD, PIK3CG, RAC2, TIAM2, TMSB4Y, VAV1*
CD28 Signaling in T Helper Cells	8.51	−3	*CD3D, CD3E, CD4, FCER1G, HLA-DOA, ITK, LCK, LCP2, PIK3CD, PIK3CG, PTPRC, VAV1, ZAP70*
Calcium-induced T Lymphocyte Apoptosis	8.11	−3	*ATP2A1, ATP2A3, CD3D, CD3E, CD4, FCER1G, HLA-DOA, LCK, PRKCB, ZAP70*

**Table 3:** Top 10 IPA canonical pathway in group B.

Ingenuity Canonical Pathways	-log (*p*-Value)	z-Score	Molecules
B1 vs. B2			
Actin Cytoskeleton Signaling	6.57	0.632	*ACTN3, ARHGAP24, EGF, FGF1, FGF10, FGF7, FGF9, HRAS, MYH1, MYH10, MYH3, MYL6B, MYLPF, PAK1, PFN2, TIAM2*
Glycolysis I	5.67	2.449	*ALDOA, ENO3, GPI, PGAM2, PGK1, PKM*
Oxidative Phosphorylation	5.17	3.162	*COX4I1, COX5A, COX7A1, CYB5A, MT-ND4L, NDUFA8, NDUFS7, NDUFS8, NDUFV1, UQCR11*
Gluconeogenesis I	4.39	2.236	*ALDOA, ENO3, GPI, PGAM2, PGK1*
Sirtuin Signaling Pathway	3.78	−1.732	*ARG2, IDH2, LDHA, MT-ND4L, NDUFA8, NDUFS7, NDUFS8, NDUFV1, PFKFB3, PGAM2, PGK1, PPIF, SREBF1, TUBA8*
Calcium Signaling	3.46	−1	*CAMKK2, CASQ2, CREB3L4, MYH1, MYH10, MYH3, MYL6B, SLC8A3, TNNI2, TNNT3, TPM1*
FGF Signaling	2.74	−0.816	*CREB3L4, FGF1, FGF10, FGF7, FGF9, HRAS*
Neuregulin Signaling	2.45	0.447	*BTC, EGF, ERBIN, HRAS, RNF41, STAT5A*
Bladder Cancer Signaling	2.43	−0.447	*EGF, FGF1, FGF10, FGF7, FGF9, HRAS*
LPS/IL-1 Mediated Inhibition of RXR Function	1.66	0.0357	*ABCB9, ACOX3, APOE, FABP4, MAOB, PLTP, SREBF1, TNFRSF11B*
B1 vs. B3			
AMPK Signaling	3.34	0.816	*ACACB, ADIPOQ, EIF4EBP1, PCK2, PFKFB3, PIK3R6, TBC1D1*
Senescence Pathway	2.07	1.633	*ACVR1C, DHCR24, E2F8, EIF4EBP1, PIK3R6, TGFB3*
Synaptogenesis Signaling Pathway	1.82	1.633	*APOE, CDH15, EIF4EBP1, GSK3B, PIK3R6, SNCG*
Factors Promoting Cardiogenesis in Vertebrates	1.8	2	*ACVR1C, BMPR1B, GSK3B, TGFB3*
Colorectal Cancer Metastasis Signaling	1.63	2	*BAX, GSK3B, PIK3R6, PTGER3, TGFB3*
Adrenomedullin signaling pathway	1.42	1	*BAX, GSK3B, GUCY2C, PIK3R6*
B2 vs. B3			
Glycolysis I	11.6	−2.121	*ALDOA, ALDOC, ENO3, GPI, PFKM, PGAM2, PGK1, PKM*
Calcium Signaling	8.23	−0.447	*ATP2A1, CAMKK2, CASQ2, CREB3L4, MYH1, MYH3, MYL2, MYL6B, PPP3CA, TNNI2, TNNT3, TPM3*
Gluconeogenesis I	7.99	−1.633	*ALDOA, ALDOC, ENO3, GPI, PGAM2, PGK1*
Semaphorin Neuronal Repulsive Signaling Pathway	4.79	−0.378	*DPYSL2, DPYSL3, MYL2, MYL6B, PAK1, PDE4D, PLXNA3*
Actin Cytoskeleton Signaling	4.2	−1.134	*ACTN3, EGF, MYH1, MYH3, MYL2, MYL6B, PAK1, SSH2*
PFKFB4 Signaling Pathway	3.74	1	*CREB3L4, GPI, PFK M, TGFB3*
HIF1α Signaling	2.76	−1.633	*ADM, EGF, GPI, LDHA, PKM, PPP3CA*
Colanic Acid Building Blocks Biosynthesis	2.49	#NUM!	*GPI, UGP2*
White Adipose Tissue Browning Pathway	2.08	1	*CAMKK2, CREB3L4, FNDC5, LDHA*
AMPK Signaling	1.97	1	*CAMKK2, CREB3L4, GYS1, PFKM, ULK1*

## Supplementary Materials

The following are available online at https://www.mdpi.com/2073-4425/11/11/1359/s1, Figure S1: Optimal number of clusters for k-means clustering, Figure S2: IPA Summary network of Group A vs. Group B, Table S1: GO biological process of up-regulated DEGs in Group A vs. Group B, Table S2: GO biological process of up-regulated DEGs in Group A sub-clusters, Table S3: GO biological process of down-regulated DEGs, Table S4: GO biological process of up-regulated DEGs in Group B sub-clusters.
