# Supplementary material for: Gene Expression Profile in Similar Tissues Using Transcriptome Sequencing Data of Whole-Body Horse Skeletal Muscle
Source: Genes (Basel). 2020 Nov 17;11(11):1359. doi: 10.3390/genes11111359 (PMC7698552; doi:10.3390/genes11111359)
Supplement: Supplementary file 1 [file genes-11-01359-s001.zip › genes-975837-supplementary/Table S1.docx]

**Table 1.** UP DEGs GO biological process in Group A *vs* Group B.

| **GO Biological Process** | **Fold Enrichment** | **FDR** | **Genes** |
| --- | --- | --- | --- |
| positive regulation of mammary gland epithelial cell proliferation (GO:0033601) | 16.26 | 4.05 × 10^-2^ | *RREB1, ZNF703, AGAP2, IQGAP3* |
| positive regulation of transcription of Notch receptor target (GO:0007221) | 11.29 | 3.38 × 10^-2^ | *NOTCH1, NOTCH4, PLXND1, NOTCH3, CREBBP* |
| activation of protein kinase A activity (GO:0034199) | 10.7 | 3.75 × 10^-2^ | *ADCY1, ADCY5, PRKACA, PRRC1, ADCY9* |
| neuronal action potential (GO:0019228) | 8.41 | 3.10 × 10^-2^ | *KCNMB2, SCN4A, CACNA1G, KCNMB2, MYH14, CACNA1H* |
| negative regulation of blood pressure (GO:0045776) | 5.81 | 4.93 × 10^-2^ | *NOS2, GPR37L1, NOS3, SMTN, ADRB2, NOS1, PPARA* |
| positive regulation of macroautophagy (GO:0016239) | 5.46 | 1.98 × 10^-2^ | *GPSM1, PINK1, FYCO1, HUWE1, ULK1, MFN2, ADRB2, LRSAM1, TSC2* |
| regulation of systemic arterial blood pressure (GO:0003073) | 4.47 | 1.84 × 10^-2^ | *NR2F2, NAV2, ECE1, PDGFB, GPR37L1, NOS3, PLCB3, SMTN, ADRB2, RASL10B, ENG* |
| regulation of muscle adaptation (GO:0043502) | 4.41 | 5.01 × 10^-2^ | *MYH7, NOTCH1, ATP2A2, PRKACA, GTF2IRD1, NOS3, FBXO32, JARID2* |
| regulation of striated muscle contraction (GO:0006942) | 4.32 | 3.55 × 10^-2^ | *MYH7, SCN4A, PKP2, ATP2A2, PRKACA, EHD3, SMTN, NOS1, FLNA, CACNA1C* |
| positive regulation of cell morphogenesis involved in differentiation (GO:0010770) | 4.06 | 3.49 × 10^-3^ | *PLXNA2, CUL7, PLXNB3, MAPT, MEGF8, RREB1, PLXND1, FGB, NTRK3, OLFM4, CUX1, PLXNA1, TRAK1, FLNA, SEMA7A, FGG* |
