# Supplementary material for: Gene Expression Profile in Similar Tissues Using Transcriptome Sequencing Data of Whole-Body Horse Skeletal Muscle
Source: Genes (Basel). 2020 Nov 17;11(11):1359. doi: 10.3390/genes11111359 (PMC7698552; doi:10.3390/genes11111359)
Supplement: Supplementary file 1 [file genes-11-01359-s001.zip › genes-975837-supplementary/Table S2.docx]

**Table 2.** UP DEGs GO biological process in Group A.

| **GO Biological Process Complete** | **Fold Enrichment** | **FDR** | **Genes** |
| --- | --- | --- | --- |
| A12 |  |  |  |
| *methylglyoxal biosynthetic process (GO:0019242)* | *> 100* | 2.19 × 10^-2^ | *GPI, TPI1* |
| *glycerophosphate shuttle (GO:0006127)* | *> 100* | 2.17 × 10^-2^ | *GPD2, GPD1* |
| *positive regulation of fast-twitch skeletal muscle fiber contraction (GO:0031448)* | *> 100* | 2.15 × 10^-2^ | *ACTN3, ATP2A1* |
| *regulation of adenylate cyclase-activating adrenergic receptor signaling pathway involved in heart process (GO:0140192)* | *> 100* | 3.39 × 10^-2^ | *ATP2B2, NOS1* |
| *glycogen catabolic process (GO:0005980)* | *66.19* | 5.47 × 10^-7^ | *PGM1, PYGM, PHKA1, PGM2L1, PHKB, PFKM* |
| *canonical glycolysis (GO:0061621)* | *66.19* | 8.19 × 10^-11^ | *PGK1, PGM2L1, ALDOA, PKM, PGAM2, GP,I TPI1, ENO3, PFKM* |
| *gluconeogenesis (GO:0006094)* | *47.49* | 4.73 × 10^-12^ | *PGM1, PGK1, GPD2, PFKFB1, , ALDOA, , PGAM2, , GPI, TPI1, CMC2, GPD1, ENO3* |
| *negative regulation of calcineurin-NFAT signaling cascade (GO:0070885)* | *39.72* | 1.60 × 10^-2^ | *ATP2B2, ACTN3, MYOZ1* |
| *fructose metabolic process (GO:0006000)* | *39.72* | 1.59 × 10^-2^ | *PFKFB1, ALDOA, PFKFB3* |
| *muscle filament sliding (GO:0030049)* | *31.35* | 2.13 × 10^-5^ | *TNNT3, MYL1, ACTN3, MYBPC2, TPM1, TNNI2* |
| *A13* |  |  |  |
| *None* |  |  |  |
| *A23* |  |  |  |
| *cardiac myofibril assembly (GO:0055003)* | *53.26* | 1.53 × 10^-2^ | *MYL2, PROX1, MYH10, FHOD3* |
| *detection of calcium ion (GO:0005513)* | *51.36* | 3.61 × 10^-2^ | *KCNMB3, KCNMB2, CASQ2* |
| *muscle system process (GO:0003012)* | *6.5* | 3.80 × 10^-2^ | *PTGER3, MYL6B, PRKG1, MYL2, CASQ2, MYOM2, SORBS1, MAP2K6* |
| *locomotion (GO:0040011)* | *3.05* | 4.09 × 10^-2^ | *DPYSL5, GPC4, DIXDC1, PRKG1, GRB14, CORO6, TRPM4, DPYSL2, ATP1A4, ARMC2, PROX1, MYH10, NRCAM, DDX4, FHOD3, , LAMB3, FOSL1* |
| *movement of cell or subcellular component (GO:0006928)* | *2.87* | 3.70 × 10^-2^ | *DPYSL5, GPC4, DIXDC1, MYL6B, , PRKG1, GRB14, CORO6, MYL2, TRPM4, DPYSL2, ATP1A4, ARMC2, PROX1, MYH10, NRCAM, DDX4, FHOD3, BAG3, LAMB3* |
