# Supplementary material for: Gene Expression Profile in Similar Tissues Using Transcriptome Sequencing Data of Whole-Body Horse Skeletal Muscle
Source: Genes (Basel). 2020 Nov 17;11(11):1359. doi: 10.3390/genes11111359 (PMC7698552; doi:10.3390/genes11111359)
Supplement: Supplementary file 1 [file genes-11-01359-s001.zip › genes-975837-supplementary/Table S4.docx]

**Table S3.** UP DEGs GO biological process in Group B.

| **GO biological process complete** | **fold Enrichment** | **FDR** | **Genes** |
| --- | --- | --- | --- |
| B12 |  |  |  |
| canonical glycolysis (GO:0061621) | 36.77 | 1.54 × 10^-6^ | *PGK1, PGM2L1, ALDOA, PKM, PGAM2, GPI, ENO3* |
| gluconeogenesis (GO:0006094) | 27.75 | 1.62 × 10^-7^ | *PC, PGM1, SLC25A11, PGK1, ALDOA, PGAM2, GPI, GPD1, ENO3* |
| high-density lipoprotein particle remodeling (GO:0034375) | 25.03 | 4.96 × 10^-2^ | *LIPE, APOE, PLTP* |
| muscle filament sliding (GO:0030049) | 22.4 | 1.78 × 10^-4^ | *MYH3, TNNT3, ACTN3, MYBPC2, TPM1, TNNI2* |
| positive regulation of steroid metabolic process (GO:0045940) | 19.56 | 1.38 × 10^-2^ | *SREBF1, ADM, FGF1, APOE* |
| regulation of oxidative phosphorylation (GO:0002082) | 18.3 | 1.63 × 10^-2^ | *COX7A1, PGK1, ACTN3, PPIF* |
| mitochondrial electron transport, NADH to ubiquinone (GO:0006120) | 17.37 | 6.28 × 10^-4^ | *NDUFA8, MT-ND4L, DLD, NDUFS8, NDUFS7, NDUFV1* |
| glycogen metabolic process (GO:0005977) | 15.76 | 1.04 × 10^-3^ | *PGM1, PYGM, PPP1R3B, PPP1R1A, NHLRC1, PGM2L1* |
| aspartate family amino acid metabolic process (GO:0009066) | 12.44 | 1.29 × 10^-2^ | *ASNS, MTHFR, DDO, GADL1, DLD* |
| glycerolipid catabolic process (GO:0046503) | 12.23 | 1.38 × 10^-2^ | *LIPG, LIPE, FABP4, PLA2G4F, GPCPD1* |
| B13 |  |  |  |
| response to extracellular stimulus (GO:0009991) | 5.91 | 7.32 × 10^-4^ | *EIF4EBP1, HLCS, PIM1, ZC3H12A, ACVR1C, ACVR1C, C2, MTHFR, PCK2, APOE, KLF10, , BCHE, SLC22A3, ADIPOQ* |
| regulation of lipid metabolic process (GO:0019216) | 5.38 | 3.09 × 10^-2^ | *RAB38, PIK3R6, CIDEA, ANGPTL4, FGF1, ACACB, G0S2, APOE, ERBB4, AVIL, ADIPOQ* |
| positive regulation of developmental process (GO:0051094) | 3.09 | 1.84 × 10^-2^ | *ROBO2, PIK3R6, PIM1, PLXNC1, ANGPTL4, FGF1, ZC3H12A, ACACB, TGFB3, RFX3, BMPR1B, ANGPT4, APOE, RASSF4, CNTN1, KLF10, ERBB4, AVIL, ADIPOQ, CDH15, CD46* |
| positive regulation of multicellular organismal process (GO:0051240) | 2.66 | 4.20 × 10^-2^ | *ROBO2, PTGER3, PIK3R6, PIM1, PLXNC1, ANGPTL4, FGF1, ZC3H12A, ACACB, TGFB3, RFX3, BMPR1B, ANGPT4, G0S2, APOE, RASSF4, CNTN1, KLF10, ERBB4, GSDMD, AVIL, ADIPOQ, CD46* |
| B23 |  |  |  |
| sarcomere organization (GO:0045214) | 30.48 | 4.17 × 10^-2^ | *LMOD2, MYOZ2, CASQ2, FHOD3* |
