# Supplementary figures and images for: Gene Expression Profile in Similar Tissues Using Transcriptome Sequencing Data of Whole-Body Horse Skeletal Muscle
Source: Genes (Basel). 2020 Nov 17;11(11):1359. doi: 10.3390/genes11111359 (PMC7698552; doi:10.3390/genes11111359)

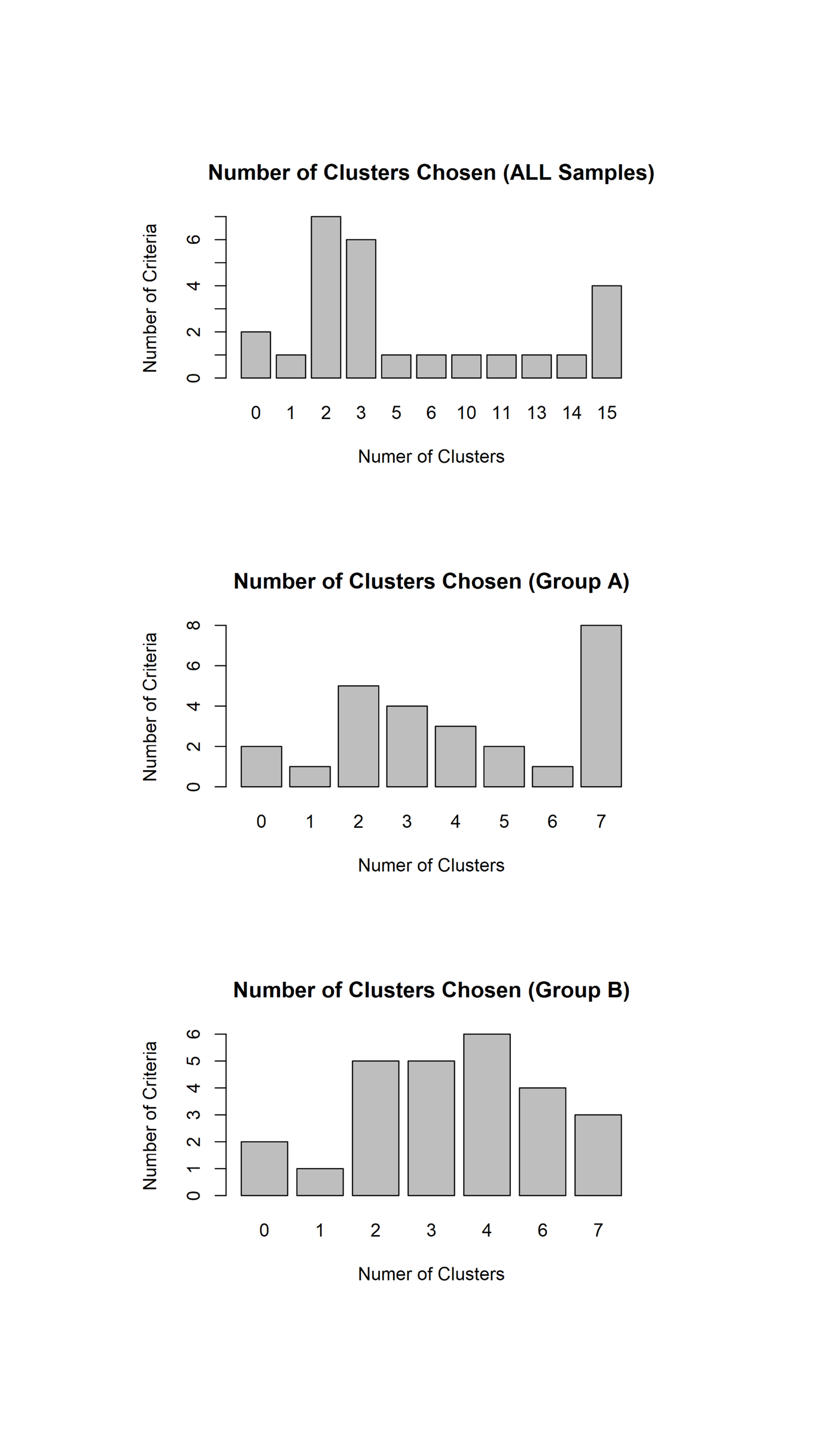

Supplement: Supplementary file 1 [file genes-11-01359-s001.zip › genes-975837-supplementary/Figure S1.tif]

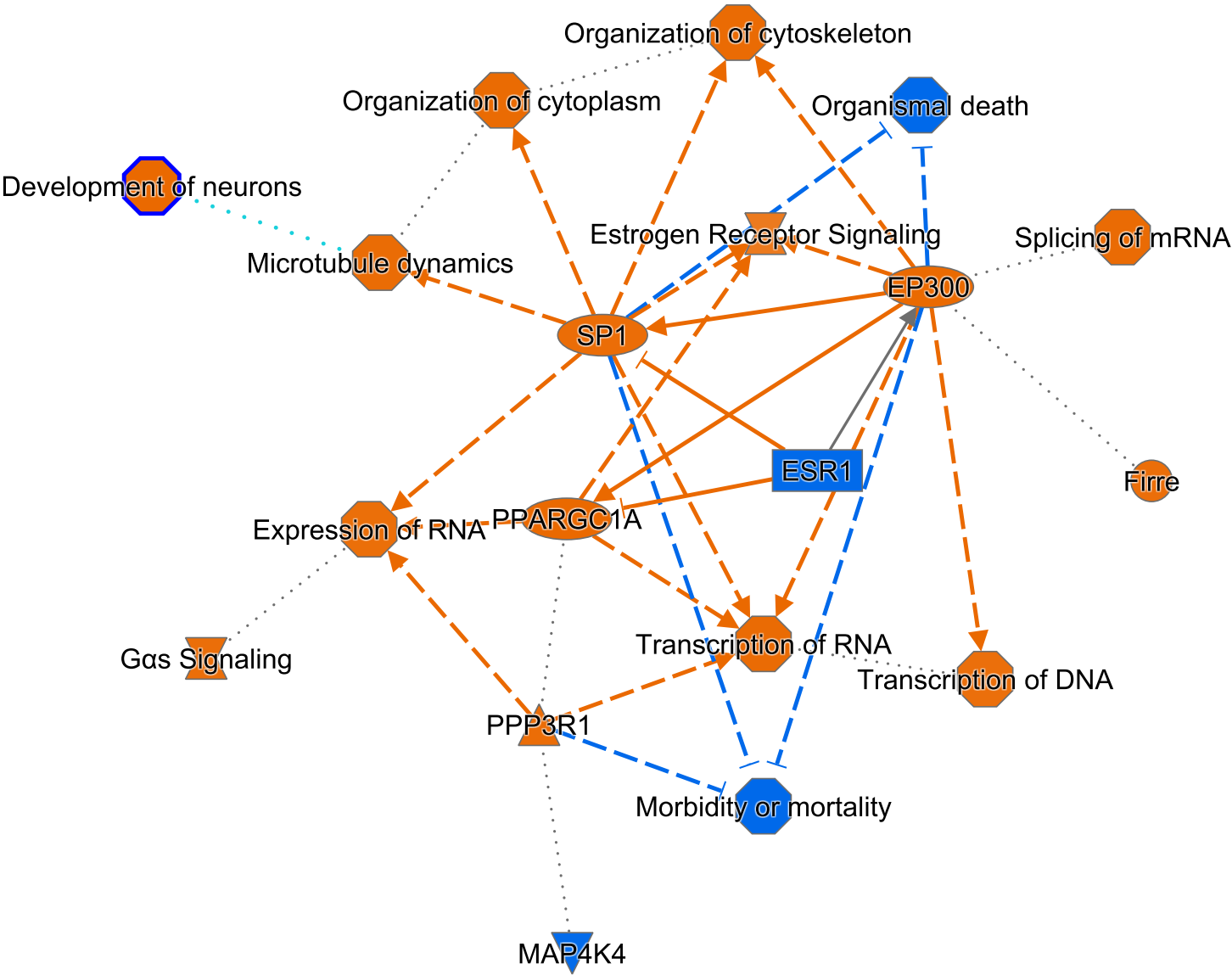

Supplement: Supplementary file 1 [file genes-11-01359-s001.zip › genes-975837-supplementary/Figure S2.pdf]
